# Impact of 13-Valent Pneumococcal Conjugate Vaccine on Colonization and Invasive Disease in Cambodian Children

**DOI:** 10.1093/cid/ciz481

**Published:** 2019-06-07

**Authors:** Paul Turner, Phana Leab, Sokeng Ly, Sena Sao, Thyl Miliya, James D Heffelfinger, Nyambat Batmunkh, Fernanda C Lessa, Jenny A Walldorf, Terri B Hyde, Vichit Ork, Md Shafiqul Hossain, Katherine A Gould, Jason Hinds, Ben S Cooper, Chanpheaktra Ngoun, Claudia Turner, Nicholas P J Day

**Affiliations:** 1 Cambodia Oxford Medical Research Unit, Angkor Hospital for Children, Siem Reap; 2 Centre for Tropical Medicine and Global Health, Nuffield Department of Medicine, University of Oxford, United Kingdom; 3 Regional Office for the Western Pacific, World Health Organization, Manila, Philippines; 4 Centers for Disease Control and Prevention, Atlanta, Georgia; 5 National Immunisation Program, Ministry of Health, Cambodia; 6 World Health Organization, Phnom Penh, Cambodia; 7 Institute for Infection and Immunity, St George’s, University of London, United Kingdom; 8 Bacterial Microarray Group at St George’s Bioscience, London Bioscience Innovation Centre, United Kingdom; 9 Mahidol-Oxford Tropical Medicine Research Unit, Faculty of Tropical Medicine, Mahidol University, Bangkok, Thailand

**Keywords:** *Streptococcus pneumoniae*, colonization, vaccine, children, Cambodia

## Abstract

**Background:**

Cambodia introduced the 13-valent pneumococcal conjugate vaccine (PCV13) in January 2015 using a 3 + 0 dosing schedule and no catch-up campaign. We investigated the effects of this introduction on pneumococcal colonization and invasive disease in children aged <5 years.

**Methods:**

There were 6 colonization surveys done between January 2014 and January 2018 in children attending the outpatient department of a nongovernmental pediatric hospital in Siem Reap. Nasopharyngeal swabs were analyzed by phenotypic and genotypic methods to detect pneumococcal serotypes and antimicrobial resistance. Invasive pneumococcal disease (IPD) data for January 2012–December 2018 were retrieved from hospital databases. Pre-PCV IPD data and pre-/post-PCV colonization data were modelled to estimate vaccine effectiveness (VE).

**Results:**

Comparing 2014 with 2016–2018, and using adjusted prevalence ratios, VE estimates for colonization were 16.6% (95% confidence interval [CI] 10.6–21.8) for all pneumococci and 39.2% (95% CI 26.7–46.1) for vaccine serotype (VT) pneumococci. There was a 26.0% (95% CI 17.7–33.0) decrease in multidrug-resistant pneumococcal colonization. The IPD incidence was estimated to have declined by 26.4% (95% CI 14.4–35.8) by 2018, with a decrease of 36.3% (95% CI 23.8–46.9) for VT IPD and an increase of 101.4% (95% CI 62.0–145.4) for non-VT IPD.

**Conclusions:**

Following PCV13 introduction into the Cambodian immunization schedule, there have been declines in VT pneumococcal colonization and disease in children aged <5 years. Modelling of dominant serotype colonization data produced plausible VE estimates.

## Teaser

The introduction of the 13-valent pneumococcal conjugate vaccine into Cambodia has led to declines in vaccine-type and antimicrobial-resistant pneumococcal colonization and disease in young children. The inclusion of multiple-serotype colonization data did not significantly alter vaccine effectiveness estimates.

**(See the Editorial Commentary by Murdoch on pages 1589–90.)**

The introduction of pneumococcal conjugate vaccines (PCVs) has significantly reduced the incidence of invasive pneumococcal disease (IPD; *Streptococcus pneumoniae* infection with a positive, sterile site culture [1]) and has led to declines in antimicrobial-resistant (AMR) IPD [2]. However, given the large number of serotypes not included in current PCV formulations, initial declines in overall IPD and AMR IPD incidences have been eroded by increases in nonvaccine serotype IPD [3, 4].

Nasopharyngeal (NP) pneumococcal colonization is common in childhood, and colonization-based surveillance may be used to predict serotype replacement and IPD incidence changes post-PCV introduction [5–8]. Children may carry multiple serotypes concurrently [9]. A decline in multiple-serotype colonization has been noted following PCV introduction [10], but the effects of multiple-serotype colonization on PCV impact models are unknown.

The uptake of PCV in Asia has been relatively slow [11]. With Gavi support, in January 2015 Cambodia added PCV13 to the national immunization schedule with a 3 + 0 dosing schedule (6, 10, and 14 weeks; no booster) and no catch-up campaign. Following the rollout, national PCV13 coverage estimates for the first and third doses were 102% (reflecting potential denominator issues) and 77% in 2015, 100% and 96% in 2016, and 93% and 91% in 2017, respectively [11].

To date, there are limited pneumococcal disease data for Cambodia. A nongovernmental pediatric hospital documented that, between 2007 and 2012, *S. pneumoniae* was responsible for ∼10% of bloodstream infections in hospitalized children, with a case fatality rate of 15.6% [12]. Pre-PCV13 introduction surveys at this hospital documented pneumococcal colonization in 68% of outpatient children aged <5 years [13].

The objective of this study was to estimate the PCV13 impact on NP colonization, invasive disease, and AMR in Cambodian children utilizing pre- and post-PCV13 introduction data from a well-established sentinel surveillance site.

## METHODS

### Study Site

Angkor Hospital for Children (AHC) is a nongovernmental pediatric hospital located in the northwestern city of Siem Reap. The hospital, and an associated satellite clinic at Sot Nikom district hospital, has ∼100 inpatient beds and provides free primary- to tertiary-level health care to children <16 years of age, without geographic restrictions. There are ∼180 000 outpatient visits and 6000 inpatient admissions per year.

### Nasopharyngeal Colonization Surveys

There were 6 discrete, outpatient-based NP colonization surveys undertaken between January 2014 and January 2018. Of these, 2 pre-PCV surveys (January and August 2014) have been described previously [13]. Pre-PCV data from children aged <5 years were further analyzed in the current study. For each of 4 post-PCV surveys (August 2015, January 2016, January 2017, and January 2018), the aim was to recruit, over a period of a month, 450 children aged <5 years who were presenting to the hospital outpatient department with minor illnesses. Children with suspected pneumonia and/or requiring hospitalization were excluded, and each child could be enrolled only once per survey. Each child’s immunization status was captured by parent/guardian recall or from the immunization record card, where available. A nasopharyngeal swab was taken from each child.

The World Health Organization (WHO) colonization detection protocol was used to identify pneumococcal serotype(s) present in the swabs [14]. Antimicrobial susceptibility testing was performed following Clinical Laboratory and Standards Institute guidelines [15]. Penicillin nonsusceptibility was defined as a minimum inhibitory concentration of ≥0.12 µg/mL. Multidrug resistance (MDR) was defined as resistance to ≥3 drug classes (Supplementary Methods).

### Detection of Multiple Pneumococcal Serotype Colonization

To determine characteristics of multiple-serotype colonization pre- and post-PCV introduction, 500 pneumococcus-positive NP swabs were further processed by latex sweep and molecular serotyping microarray methods [9]. There were 100 pneumococcus-positive swabs selected randomly from each January survey, except for 2018, where the first 100 eligible swabs were selected (Supplementary Methods).

### Invasive Pneumococcal Disease Data

Culture-confirmed IPD cases from 1 January 2012 to 31 December 2018 were identified from the hospital laboratory database, which captures data on all clinical specimens submitted for culture. A case of IPD was defined as having *S. pneumoniae* isolated from blood, cerebrospinal fluid, or other normally sterile sites in a child aged <5 years of age. Only the first isolate from each infection episode was included. Over this time period, blood and other syndrome-appropriate specimens were taken for culture on children requiring hospitalization with fever and/or signs of sepsis, at the discretion of the treating clinician. Specimen collection guidelines and active diagnostic stewardship were available throughout [16]. Details of specimen processing have been summarized elsewhere [17].

### Data Analysis

Categorical variables were compared by the Chi-squared or Fisher’s exact test. Trends were assessed by the Cochran-Armitage test. Non–normally distributed continuous variables were compared by the Wilcoxon rank sum or Kruskal-Wallis test. Analyses were done using R version 3.5.1 [18].

An estimation of PCV13’s effect was done by an assessment of changes in the vaccine-type (VT; serotypes 1, 3, 4, 5, 6A, 6B, 7F, 9V, 14, 18C, 19F, 19A, and 23F) pneumococcal colonization prevalence pre-PCV introduction (2014) and post-PCV introduction (2016–18), stratified by age. Log-binomial regression was used to determine prevalence ratios and 95% confidence intervals (CI) for overall, VT, non–vaccine type (NVT), and nontypeable (NT) pneumococcal colonization. The models were adjusted for epidemiologic factors associated with variability in colonization: upper respiratory tract infection symptoms, cohabitation with another child aged <5 years, definite recent antibiotic use, and enrollment season (January vs August) [19–21]. Vaccine effectiveness (VE) was calculated as 100 × (1 minus the adjusted prevalence ratio).

IPD detection rates were estimated using the total number of blood cultures processed from children aged <5 years as the denominator, since the hospital catchment area cannot be readily quantified and this denominator accounts for temporal variations in culture practices. Poisson regression was used to determine incidence rate ratios for invasive disease.

Modelling of vaccine effects using combined colonization and disease data was done as specified by Weinberger et al (their Model 1, see Supplementary Methods) [7].

### Ethics Statement

Colonization survey protocols were approved by the AHC Institutional Review Board (422/13, 371/14, and 0348/15), the Cambodia National Ethics Committee for Health Research (210NECHR, 289NECHR, 150NECHR, and 137NECHR), the WHO Western Pacific Regional Office Institutional Review Board (2015.6.CAM.1.EPI), and the University of Oxford Tropical Research Ethics Committee (1009–13 and 559-15). The analysis of stored swabs by latex sweep and microarray was determined by the US Centers for Disease Control and Prevention Center for Global Health Human Subjects Review to be a nonresearch activity (CGH HSR 2017–532), and a Centers for Disease Control and Prevention Institutional Review Board review was not required.

## RESULTS

### Study Participants

A total of 1805 NP swabs were collected in the 4 post-PCV surveys. There were 4 swabs that were removed due to enrollment errors, leaving 1801 analyzable swabs. There were 721 NP swabs collected from children aged <5 years in the 2 pre-PCV surveys, giving a combined total of 2522 NP swabs.

The median age of children was 1.51 years (interquartile range [IQR] 0.76–2.88) in the pre-PCV surveys and 1.39 years (IQR 0.77–2.64) in the post-PCV surveys (*P* = .2). There were 3 children (0.1%) who were known to be living with human immunodeficiency virus. Demographic and basic health data on the children contributing swabs are summarized in Table 1. Half (52.7%, 949/1801) of the children enrolled in post-PCV surveys were age-eligible to have been fully immunized: 86.7% (827/949) of these children were reported to have received ≥2 doses of PCV13. In the 2018 survey, ≥2 doses of PCV13 had been received by 75.4% (342/453) of enrolled children, although this was verified by vaccine record visualization in only 65 children (14.3%).

**Table 1.** Demographic, Health, and 13-Valent Pneumococcal Conjugate Vaccine Immunization Summary Data

Variable	Pre-PCV		Post-PCV				*P* Value
	Jan 2014	Aug 2014	Aug 2015	Jan 2016	Jan 2017	Jan 2018	
Children, n	373	348	450	449	449	453	…
Demographic/health							
Age in years, median (IQR)	1.35 (0.73–2.78)	1.46 (0.79–2.95)	1.51 (0.82–2.79)	1.26 (0.75–2.40)	1.49 (0.77–2.90)	1.36 (0.76–2.38)	.1
Gender, n (% female)	165 (44.2)	171 (49.1)	222 (49.3)	218 (48.6)	215 (47.9)	215 (47.5)	.7
URTI symptoms, n (%)	352 (94.4)	265 (76.1)	426 (94.7)	422 (94.0)	402 (89.5)	404 (89.2)	.5
Cohabiting with other children <5 years, n (%)	107 (28.8)	102 (29.3)	162 (36.0)	134 (29.9)	156 (34.7)	156 (34.4)	.06
Definite recent antibiotic use, n (%)	13/371 (3.5)	24/346^a^ (6.9)	27 (6.0)	11/447^a^ (2.5)	12 (2.7)	15 (3.3)	.03
PCV immunization							
PCV13, ≥1 dose, verified,^b^ n (%)	0 (0.0)	0 (0.0)	25 (5.6)	49 (10.9)	51 (11.4)	72 (15.9)	…
PCV13, ≥1 dose, parent recall, n (%)	0 (0.0)	0 (0.0)	41 (9.1)	133 (29.6)	222 (49.7)	291 (64.2)	…
PCV13, ≥2 doses, verified,^b^ n (%)	0 (0.0)	0 (0.0)	24 (5.3)	42 (9.4)	46 (10.3)	65 (14.3)	…
PCV13, ≥2 doses, parent recall, n (%)	0 (0.0)	0 (0.0)	40 (8.9)	130 (29.0)	204 (45.6)	277 (61.1)	…

Data are for children included in the pre- and post-PCV colonization surveys.

Abbreviations: PCV13, 13-valent pneumococcal conjugate vaccine; IQR, interquartile range; PCV, pneumococcal conjugate vaccine; URTI, upper respiratory tract infection.

^a^Data missing for 2 cases in August 2014 and January 2016 surveys.

^b^Immunization status verified by visualization of the child’s personal immunization record.

### Pneumococcal Colonization

**Figure 1.**
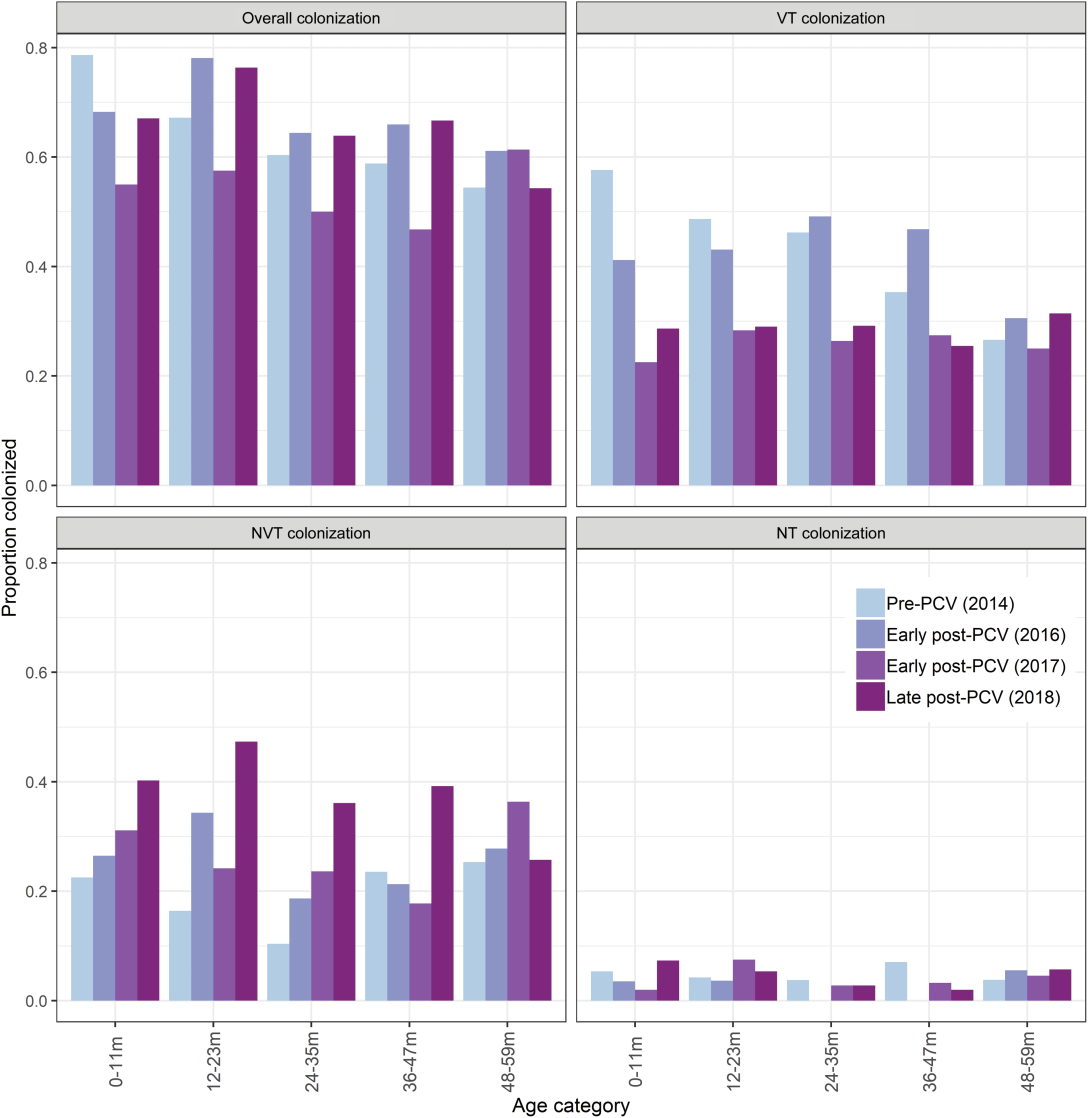
Pneumococcal colonization, stratified by age category, pneumococcal serotype category, and time period. Results for 2014 are the combined data from January and August surveys. Abbreviations: NT, nontypeable; NVT, nonvaccine type; PCV, pneumococcal conjugate vaccine; VT, vaccine type.

Two-thirds (1629/2522, 64.6%) of children were colonized by *S. pneumoniae*: 68.0% (490/721) in the pre-PCV surveys and 63.2% (1139/1801) in the post-PCV surveys (*P* = .03). Comparing the pre-PCV time period with the early post-PCV and late post-PCV time periods, the overall colonization prevalence and colonization by penicillin nonsusceptible/MDR pneumococci decreased substantially in the children aged 0–11 months (Figure 1; Supplementary Table S1). There were declines in colonization by VT pneumococci and increases in NVT colonization in the 0–11 month, 12–23 month, and 24–35 month age groups. No clear changes in colonization characteristics were noted in the 36–47 month and 48–59 month age groups, who were not age-eligible for PCV13 (Figure 1; Supplementary Table S1).

**Figure 2.**
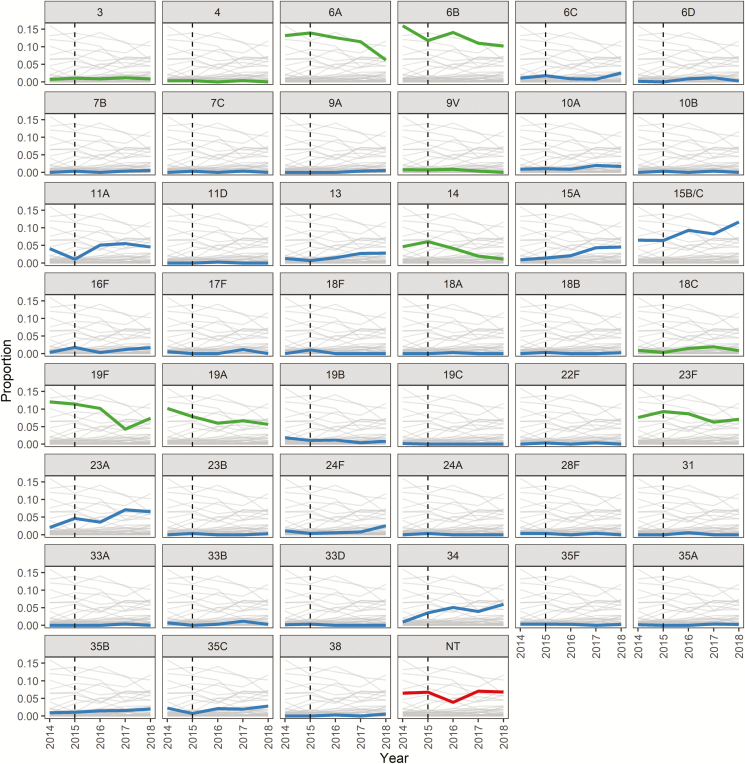
Pneumococcal serotype colonization, by proportion of total isolates in each time period. Light gray lines show detected serotypes as a proportion of all isolates from that year, with a single serotype highlighted in color (green = vaccine type; blue = nonvaccine type; red = nontypeable [NT]). The vertical black dashed line represents 13-valent pneumococcal conjugate vaccine introduction.

NP swab cultures yielded 1759 pneumococci. There were 45 serotypes plus NT isolates identified: 56.1% of isolates were VT. Serotypes 6A and 6B were the most commonly carried serotypes in all surveys until January 2018, when serotypes 15B/C dominated (Supplementary Figure S1). The rates of VT isolates decreased, NVT isolates increased (notably 15A, 15B/C, 23A, and 34), and NT isolates did not change over time (Figure 2). Penicillin, co-trimoxazole, and tetracycline resistance rates were high (>60%), with lower rates of resistance to macrolides, ceftriaxone, and chloramphenicol (Supplementary Table S2). VT isolates were more likely to be MDR than NVT or NT isolates (86.8% vs 47.6%, respectively; *P* < .001). The proportion of isolates that were penicillin nonsusceptible decreased over time (81.0% in 2014 to 65.6% in 2018; *P* < .0001), with smaller declines in tetracycline resistance and MDR. There were no clear trends in the MDR prevalences within serotype categories over time (Supplementary Figure S2).

### Multiple Pneumococcal Serotype Colonization

In the 500 cases selected for detailed analyses, multiple pneumococcal serotype colonization was detected in 10.0% (50/500), 12.2% (61/500), and 20.8% (104/500) of children by WHO serotyping, latex sweep, and microarray, respectively, with no evidence of a temporal trend (Supplementary Figure S3). Cocolonization patterns are summarized in Supplementary Figure S4.

### Invasive Disease

Between 1 January 2012 and 31 December 2018, there were 81 invasive pneumococcal disease episodes, caused by 16 serotypes, in hospitalized children aged <5 years (Supplementary Figure S5). In these episodes, 73 children had a positive blood culture alone, 5 had a positive pleural fluid culture (+ positive blood culture in 4), and 3 had a positive cerebrospinal fluid culture (+ positive blood culture in 2). The median age at presentation was 1.6 years (IQR 0.9–2.3), with no age difference between the pre- and post-PCV periods. Vaccine serotypes (italics) accounted for 91.4% (74/81) of infections: *6B* (14, 17.3%), *14* (14, 17.3%), *19A* (13, 16.0%), *1* (10, 12.3%), *23F* (6, 7.4%), *6A* (5, 6.2%), *19F* (5, 6.2%), *18C* (3, 3.7%), *3* (2, 2.5%), 12F (2, 2.5%), 38 (2, 2.5%), *4* (1, 1.2%), *5* (1, 1.2%), 13 (1, 1.3%), 15A (1, 1.2%), 23A (1, 1.2%). In the pre-PCV period, 92.9% (39/42) isolates were VT, compared with 86.4% (19/22) in the post-PCV period (*P* = 0.4; Supplementary Figures S5 and S6). The IPD detection rate fell from 3.76 (95% CI 2.71–5.08)/1000 blood cultures in the pre-PCV period to 2.33 (95% CI 1.46–3.53)/1000 blood cultures in the post-PCV period (Table 2; Supplementary Figure S7).

**Table 2.** Summary of Invasive Pneumococcal Disease Episodes per 1000 Blood Cultures in Hospitalized Children Aged <5 Years, by Time Period

	Blood cultures (n)	IPD (n)	IPD detection rate (95% CI)	VT (n)	VT detection rate (95% CI)	NVT (n)	NVT detection rate (95% CI)
Pre-PCV (2012–2014)	11 170	42	3.76 (2.71–5.08)	39	3.49 (2.48–4.77)	3	0.27 (.05–.78)
Post-PCV (2016–2018)	9425	22	2.33 (1.46–3.53)	19	2.01 (1.21–3.15)	3	0.32 (.07–.09)
Early post-PCV (2016–2017)	6556	16	2.44 (1.40–3.96)	15	2.29 (1.28–3.77)	1	0.15 (.04–.85)
Late post-PCV (2018)	2869	6	2.09 (.77–4.55)	4	1.39 (.38–3.57)	2	0.70 (.08–2.51)

Abbreviations: CI, confidence interval; IPD, invasive pneumococcal disease; NVT, nonvaccine type; PCV, pneumococcal conjugate vaccine; VT, vaccine type.

Overall, 70.4% (57/81) of invasive isolates were penicillin nonsusceptible and 67.9% (55/81) were MDR, with no change post-PCV introduction. VT pneumococci were more likely to be MDR than NVT isolates (71.4% vs 28.6%, respectively; *P* = .03).

### Estimates of 13-Valent Pneumococcal Conjugate Vaccine Effectiveness

#### Vaccine Effectiveness Against Colonization

Comparing pre- and post-PCV periods, the VE estimates for colonization were 16.6% (95% CI 10.6–21.8%) for all pneumococci and 39.2% (95% CI 26.7–46.1%) for VT pneumococci, with a 23.3% (95% CI −2.6 to 50.1%) increase in NVT colonization (Table 3). For colonization by AMR pneumococci, VE estimates were 22.5% (95% CI 15.0–29.0%; penicillin nonsusceptible) and 26.0% (95% CI 17.7–33.0%; MDR).

**Table 3.** Estimates of 13-Valent Pneumococcal Conjugate Vaccine Effectiveness Against Colonization in Cambodian Children Aged <5 Years

	Colonization Prevalence (95% CI)	Crude Prevalence Ratio (95% CI)	Adjusted^a^ Prevalence Ratio (95% CI)	*P* Value
All pneumococci				
Pre-PCV (2014)^b^	68.0 (64.4–71.4)	…	…	…
Post-PCV (2016–2018)	64.2 (61.5–66.7)	0.944 (.866–1.008)	0.834 (.782–.894)	<.0001
Early post-PCV (2016–2017)	62.1 (58.9–65.3)	0.914 (.851–.982)	0.806 (.750–.869)	<.0001
Late post-PCV (2018)	68.2 (63.7–72.5)	1.004 (.925–1.087)	0.890 (.820–.966)	.006
VT pneumococci				
Pre-PCV (2014)	47.6 (43.9–51.3)	…	…	…
Post-PCV (2016–2018)	32.3 (29.8–34.8)	0.678 (.609–.757)	0.608 (.539–.689)	<.0001
Early post-PCV (2016–2017)	34.1 (31.0–37.3)	0.716 (.636–.806)	0.642 (.563–.733)	<.0001
Late post-PCV (2018)	28.7 (24.6–33.1)	0.603 (.510–.708)	0.543 (.455–.644)	<.0001
NVT pneumococci				
Pre-PCV (2014)	19.6 (16.7–22.6)	…	…	…
Post-PCV (2016–2018)	31.5 (29.1–34.1)	1.612 (1.369–1.914)	1.233 (1.026–1.501)	.03
Early post-PCV (2016–2017)	27.1 (24.2–30.1)	1.384 (1.155–1.666)	1.056 (.865–1.302)	.6
Late post-PCV (2018)	40.4 (35.8–45.1)	2.066 (1.718–2.492)	1.587 (1.297–1.961)	<.0001
NT pneumococci				
Pre-PCV (2014)	4.9 (3.4–6.7)	…	…	…
Post-PCV (2016–2018)	4.1 (3.1–5.3)	0.839 (.557–1.280)	0.970 (.577–1.731)	.9
Early post-PCV (2016–2017)	3.5 (2.4–4.9)	0.711 (.441–1.142)	0.819 (.460–1.517)	.5
Late post-PCV (2018)	5.3 (3.4–7.8)	1.091 (.650–1.800)	1.275 (.693–2.4414)	.4
Penicillin-NS pneumococci				
Pre-PCV (2014)	57.3 (53.6–60.9)	…	…	…
Post-PCV (2016–2018)	50.7 (48.0–53.4)	0.885 (.816–.961)	0.775 (.710–.850)	<.0001
Early post-PCV (2016–2017)	51.8 (48.5–55.1)	0.904 (.827–.989)	0.791 (.719–.872)	<.0001
Late post-PCV (2018)	48.6 (43.9–53.3)	0.848 (.755–.948)	0.744 (.660–.838)	<.0001
MDR pneumococci				
Pre-PCV (2014)	51.0 (47.3–54.7)	…	…	…
Post-PCV (2016–2018)	44.3 (41.7–47.0)	0.867 (.792–.954)	0.740 (.670–.823)	<.0001
Early post-PCV (2016–2017)	44.1 (40.8–47.4)	0.864 (.780–.958)	0.735 (.659–.823)	<.0001
Late post-PCV (2018)	44.8 (40.2–49.5)	0.878 (.773–.993)	0.751 (.658–.856)	<.0001

Abbreviations: CI, confidence interval; MDR, multidrug resistant; NS, nonsusceptible; NT, nontypeable; NVT, nonvaccine type; PCV, pneumococcal conjugate vaccine; VT, vaccine type.

^a^Adjusting for the presence of upper respiratory tract infection symptoms, cohabitation with a child <5 years, definite recent antibiotic use, and enrollment season (January vs August).

^b^Pre-PCV data consist of 2 colonization surveys from January and August 2014.

#### Vaccine Effectiveness Against Invasive Disease

There was a 37.9% (95% CI 63.6 to −2.9%) decline in overall IPD (*P* = .07) and a 42.3% (95% CI 67.3 to −1.5%) decline in VT IPD (*P* = .05) between the pre- and post-PCV periods. No overall change in NVT IPD was detected (*P* = .8; Table 4).

**Table 4.** Observed Estimates of 13-Valent Pneumococcal Conjugate Vaccine Effectiveness

	IRR (95% CI)	Change in disease rate, % (95% CI)
All IPD		
Post-PCV (2016–2018)	0.621 (.364–1.029)	−37.9 (−63.6 to 2.9)
Early (2016–2017)	0.649 (.354–1.131)	−35.1 (−64.6 to 13.1)
Late (2018)	0.556 (.212–1.211)	−44.4 (−78.8 to 21.1)
VT IPD		
Post-PCV (2016–2018)	0.577 (.327–.985)	−42.3 (−67.3 to 1.5)
Early (2016–2017)	0.655 (.350–1.163)	−34.5 (−64.9 to 16.3)
Late (2018)	0.399 (.120–.992)	−60.1 (−88.0 to .8)
NVT IPD		
Post-PCV (2016–2018)	1.185 (.219–6.404)	18.5 (−78.1 to 540.4)
Early (2016–2017)	0.568 (.028–4.435)	−43.2 (−97.2 to 343.5)
Late (2018)	2.596 (.342–15.666)	159.6 (−65.8 to 1466.6)

Based on pre- and post-PCV IPD data in hospitalized children aged <5 years, 1 January 2012 to 31 December 2018.

Abbreviations: CI, confidence interval; IPD, invasive pneumococcal disease; IRR, incidence rate ratio; NVT, nonvaccine type; VT, vaccine type.

#### Combined Colonization–invasive Disease Model Estimates Of Vaccine Effectiveness Against Invasive Disease

**Figure 3.**
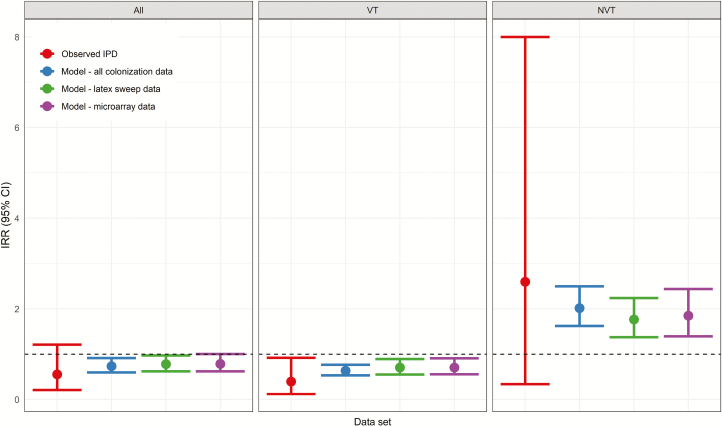
Observed and modeled IPD IRRs for the late post-PCV (2018) and pre-PCV (2012–2014) time periods. For clarity, the upper bound of the confidence interval for the observed NVT IRR has been truncated at 8 (actual value 15.6). The dashed horizontal line indicates an IRR of 1. Abbreviations: CI, confidence interval; IPD, invasive pneumococcal disease; IRR, incidence rate ratio; NVT, nonvaccine type; PCV, pneumococcal conjugate vaccine; VT, vaccine type.

In the model including pre-PCV IPD data and the pre- and post-PCV colonization data, the IPD incidence was estimated to have declined by 26.4% (95% CI 14.4–35.8) in 2018 (Supplementary Table S3). The VT IPD incidence was estimated to have declined by 36.3% (95% CI 23.8–46.9), with a 101.4% (95% CI 62.0–145.4) increase in NVT IPD. Repeating the model with a subset of carriage data and sensitive methods to detect multiple-serotype colonization data yielded VE estimates that were very similar to each other (Supplementary Table S4). The point estimates were all slightly decreased compared with the model, including the entire colonization data set processed to detect dominant serotype(s) only (Figure 3).

## DISCUSSION

This study describes the effects of PCV13 on pneumococcal colonization, invasive disease, and AMR in Cambodian children 3 years after its introduction into the routine immunization schedule.

PCV13 was introduced without a catch-up campaign. However, coverage of target children was reported to be high nationally and, by the 2018 colonization survey, around three-quarters of enrolled children <5 years were reported to have received ≥2 doses. By 3 years following introduction, there was a 39% decline in VT colonization and a 23% increase in NVT colonization. Despite declines, the prevalence of VT colonization among children age-eligible to have received PCV13 continued to be high (~29%), which may explain the lack of indirect effects observed in children >36 months: that is, those too old to have received PVC13. In comparison, 2 years after the introduction of PCV10 in Kilifi, Kenya (with a catch-up campaign), the VT colonization prevalence had declined by 64% and NVT colonization had increased by 37% in children aged <5 years [21]. Reductions in VT colonization of 44–66% and increases in NVT colonization of 5–72% were reported recently in young children 3 years after PCV10 introduction in Fiji, an upper middle-income country in the Asia-Pacific region [22]. A recent model-based study estimated that it would take approximately 10 years to eliminate VT colonization, with almost complete replacement by NVT colonization, if PCV13 were introduced in Vietnam without a catch-up campaign [23]. In due course, it will be important to compare findings from our study to ongoing colonization-based PCV impact studies in Lao People’s Democratic Republic and Papua New Guinea [24].

Antimicrobial resistance rates were high in VT pneumococci and reductions in colonization by MDR (VE 26%) and penicillin nonsusceptible pneumococci (VE 23%) was seen following the PCV13 introduction. At the isolate level, there was a decline in the proportion of pneumococci that were penicillin nonsusceptible, from 81% in 2014 to 66% in 2018.

The nature of the study site meant that an estimation of population IPD rates was not possible. However, comparing post-PCV (January 2016 to December 2018) with pre-PCV (January 2012 to December 2014) blood culture data, a 38% decline in overall IPD was detected, with a 42% decline in VT IPD. Unfortunately, the small number of positive cultures and wide confidence intervals limit the interpretability of these results. This is a frequent problem when attempting to estimate the impact of PCVs in low- and middle-income countries, where microbiology and epidemiologic surveillance resources are scarce and prehospital treatment may reduce blood culture yields [25]. However, it has been shown that reasonable estimates of PCV impacts could be obtained by modelling pre-PCV IPD data with changes in colonization before and after PCV7 introduction [7]. Using the data from the current study, this approach estimated that there would have been a 26% decrease in overall IPD, a 36% decrease in VT IPD, and a 101% increase in NVT IPD incidences in 2018, compared to baseline (2012–2014). These point estimates are somewhat more modest than the post-PCV declines in IPD observed in large, population-based IPD surveillance in other low- and middle-income countries [26, 27], perhaps as a result of the known limitations of the model, which tends to underestimate declines in VT disease as a result of the requirement for the inclusion of continuity corrections for noncarried serotypes. Weinberger and colleagues [7] were cautiously optimistic that their PCV7-validated model would produce reasonable results when applied to PCV13 data, but stressed that validation would be important using data sets where temporally variable and highly invasive serotypes, such as 1 and 5, are found in NP specimens. We identified 45 colonizing serotypes and 16 serotypes from IPD cases. Serotype 1 was responsible for 13% of IPD cases, and disappeared rapidly after PCV13 introduction, but was not detected in NP specimens. Whilst we cannot formally validate the model results, the findings demonstrate comparable trends to those from population-based surveillance, and the modelled confidence intervals overlap with those from population-based studies in other locations. However, given the heterogeneity in invasiveness between serotypes, ongoing IPD surveillance remains a critically important activity for monitoring the vaccine impacts [28].

The impact of inclusion of multiple–pneumococcal serotype colonization data on disease model estimates was unknown, and at least 1 study has demonstrated a decline in multiple-serotype colonization following PCV introduction [10]. In Cambodian children, multiple-serotype colonization was detected in up to 24% of NP swabs using sensitive methodologies, but did not vary significantly over time. The inclusion of multiple serotype data did not impact on modelled VE estimates, suggesting that dominant serotype data generated from studies using the standard WHO methodology for pneumococcal colonization detection is adequate for the determination of VE in such models [14].

The study has limitations. All data came from a single sentinel surveillance site, which may limit generalizability. However, given the sample size, location, and unrestricted catchment area (where approximately one-third of patients reside outside of Siem Reap province), the study population is likely to be representative of many children in Cambodia. The selection of children with minor illnesses only was done to minimize potential biases of recruiting hospital attendees into the colonization surveys. The small number of children in whom PCV immunization statuses could be verified from their personal immunization record cards meant that planned analyses of PCV’s impact on colonization at the individual level were not possible. The lack of a population denominator limits the utility of IPD data. However, this has been compensated by estimating VE using a combined colonization–invasive disease data approach, which was previously well validated for the estimation of VE for PCV7 [7]. The frequency of blood culture collections in the AHC outpatient department decreased significantly in late 2015 following the introduction of updated laboratory guidelines, which may have reduced IPD case detection rates, although there were no changes in culture practices for patients requiring hospitalization. This change in diagnostic practice will not have impacted on modelled VE estimates. Finally, only 3 years of post-PCV data were collected. Future surveillance efforts should ideally include older children and adults to capture the indirect effects of immunization.

In conclusion, the introduction of PCV13 into the childhood immunization schedule in Cambodia has resulted in declines in VT pneumococcal colonization and disease in children aged <5 years. Ongoing surveillance will be critical to determine further changes as the PCV13 immunization program matures.

## Supplementary Data

Supplementary materials are available at *Clinical Infectious Diseases* online. Consisting of data provided by the authors to benefit the reader, the posted materials are not copyedited and are the sole responsibility of the authors, so questions or comments should be addressed to the corresponding author.

ciz481_suppl_Supplement_Material

## Notes

***Acknowledgments.*** The authors thank the patients and their parents and the staff of Angkor Hospital for Children.

***Financial support.*** This work was supported by grants from the World Health Organization, US Centers for Disease Control and Prevention, and the Li Ka Shing Global Health Foundation–University of Oxford Programme. The Angkor Hospital for Children Microbiology Laboratory is supported by the Kadoorie Charitable Foundation. Cambodia Oxford Medical Research Unit is part of the Mahidol-Oxford Tropical Medicine Research Unit Tropical Health Network and is funded by Wellcome (grant number 106698/Z/14/Z). B. S. C. was supported by The Medical Research Council and Department for International Development (grant number MR/K006924/1).

***Potential conflicts of interest.*** J. H.’s institution (St George’s, University of London [SGUL]) has received funding from GSK, Sanofi Pasteur, and Pfizer for research conducted by J. H. J. H. is a cofounder, board member, and shareholder of Bacterial Microarray Group at St George’s (BUGS) Bioscience, a not-for-profit spin-out company of SGUL, but J. H. receives no personal income from this organization. K. A. G.’s institution (SGUL) subcontracted K. A. G. to BUGS Bioscience, but K. A. G. receives no personal income from this activity. J. D. H. owns stock in Pfizer. All other authors report no potential conflicts. All authors have submitted the ICMJE Form for Disclosure of Potential Conflicts of Interest. Conflicts that the editors consider relevant to the content of the manuscript have been disclosed.
