# Supplementary material for: Impact of 13-Valent Pneumococcal Conjugate Vaccine on Colonization and Invasive Disease in Cambodian Children
Source: Clin Infect Dis. 2019 Jun 7;70(8):1580–8. doi: 10.1093/cid/ciz481 (PMC7145996; doi:10.1093/cid/ciz481)
Supplement: ciz481_suppl_Supplement_Material [file ciz481_suppl_supplement_material.pdf]

Supplementary Material

Impact of 13-Valent Pneumococcal Conjugate Vaccine on Colonisation and Invasive Disease in Cambodian Children

Paul Turner[1,2], Phana Leab[1], Sokeng Ly[1], Sena Sao[1], Thyl Miliya[1], James D. Heffelfinger[3], Nyambat Batmunkh[3], Fernanda C. Lessa[4], Jenny A. Walldorf[4], Terri B. Hyde[4], Vichit Ork[5], Md. Shafiqul Hossain[6], Katherine A. Gould[7,8], Jason Hinds[7,8], Ben S. Cooper[2,9], Chanpheaktra Ngoun[1], Claudia Turner[1,2], Nicholas P.J. Day[2,9]

Affiliations

[1]Cambodia Oxford Medical Research Unit, Angkor Hospital for Children, Siem Reap, Cambodia

[2]Centre for Tropical Medicine and Global Health, Nuffield Department of Medicine, University of Oxford, Oxford, UK

[3]Regional Office for the Western Pacific, World Health Organization, Manila, Philippines

[4]Centers for Disease Control and Prevention, Atlanta, GA, USA

[5]National Immunisation Program, Ministry of Health, Phnom Penh, Cambodia

[6]World Health Organization, Phnom Penh, Cambodia

[7]Institute for Infection and Immunity, St George's, University of London, UK

[8]BUGS Bioscience, London Bioscience Innovation Centre, London, UK

[9]Mahidol-Oxford Tropical Medicine Research Unit, Faculty of Tropical Medicine, Mahidol University, Bangkok, Thailand

Methods

Nasopharyngeal swab collection

Each child enrolled into a colonisation survey had a flocked nylon nasopharyngeal swab taken at recruitment (Medical Wire and Equipment, Corsham, UK). Swabs were stored immediately in 1ml STGG medium (skim milk-tryptone-glucose-glycerol; prepared in-house) in a cool box and frozen at -80°C within eight hours prior to further processing [1].

Primary detection of pneumococcal colonisation

All morphologically distinct suspected pneumococci, i.e. colonies with alpha haemolysis on 5% sheep blood agar supplemented with 5mg/L gentamicin (Columbia agar base, Oxoid, Basingstoke, UK; prepared in-house), were identified by optochin susceptibility with bile solubility confirmation of intermediate results [1]. Serotypes were determined by latex agglutination and/or Quellung reaction, as described previously [2]. Non-typeable (NT) isolates were confirmed by bile solubility and a negative capsular swelling reaction using Omniserum (SSI Diagnostica, Hillerød, Denmark). Pneumococcal characterisation was quality assured by participation in the UK NEQAS / WHO IB-VPD EQA scheme (laboratory identification number: WH244).

Antimicrobial susceptibility testing of pneumococcal isolates

Antimicrobial susceptibility testing (AST) was performed for all isolates, according to Clinical Laboratory and Standards Institute (CLSI) guidelines [3]. However, citrated sheep blood, rather than defibrinated, was used to prepare 5% blood Mueller-Hinton agar plates. Breakpoints from the 2018 version of the CLSI guidelines were used to determine AST categories for benzylpenicillin (Etest minimum inhibitory concentration (MIC); bioMerieux, Marcy-l'Etoile, France), ceftriaxone (Etest MIC), chloramphenicol (disk diffusion; Oxoid), clindamycin (disk diffusion), erythromycin (disk diffusion), co-trimoxazole (disk diffusion), and tetracycline (disk diffusion) [4]. Resistance to penicillin and ceftriaxone was defined as an MIC of ≥0.12 µg/mL and ≥1 µg/mL, respectively. For the other agents, the "intermediate" resistance category was re-classified as "susceptible" to give a conservative estimate of resistance for agents where MIC values were not measured. Multi-drug resistance (MDR) was defined as resistance to three or more agents, with clindamycin/erythromycin and benzylpenicillin/ceftriaxone counting as single agents.

Citrated 5% sheep blood Mueller Hinton agar and antimicrobial disks were quality controlled (QC) weekly and Etest strips underwent monthly QC using *S. pneumoniae* ATCC 46919. Verification of a subset of 100 AST results was done, using defibrinated sheep blood Mueller Hinton agar to retest the isolates, with >95% categorical concordance [5-7].

Identification of multiple pneumococcal serotype colonisation

Swabs were eligible for inclusion if the child's residence was in Siem Reap province and at least a moderate growth of pneumococcus was detected in the primary swab culture (≥2+ on a semi-quantitative 0 – 4+ scale). After thawing, 100µl NPS-STGG was streaked onto two agar 5%

sheep blood - gentamicin agar plates. Following overnight incubation at 36°C in 5% CO_2 the colonies from each plate were prepared for latex sweep and microarray-based molecular serotyping, respectively. All colonies from the first plate were suspended in 0.5ml sterile 0.85% saline and the density adjusted to 2-3 McFarland by addition of further saline. Serotypes contained in the suspension were determined by reactions with specific pool, group, type, and factor latex antisera, as previously described [8]. One millilitre of sterile phosphate buffered saline was pipetted onto the surface of the second blood agar plate and all colonies were emulsified using a sterile loop. This suspension was transferred to a sterile 1.5ml tube and centrifuged for five minutes to pellet the bacterial cells. Following centrifugation, the supernatant was removed and the cells stored at -80°C prior to further processing in the UK. DNA extraction was performed using a QIAcube HT instrument (Qiagen, Hilden, Germany), using a Gram positive bacteria protocol. Molecular serotyping and antimicrobial resistance detection by microarray were done using Senti-SP v1.7 (BUGS Bioscience, London, UK), as described elsewhere [9, 10].

Modelling of vaccine effectiveness using combined colonisation and disease data [11]

Pre- and post-PCV serotype colonisation prevalence were determined (number of serotype positive swabs divided by the number of swabs collected) and the average change (inverse variance-weighted) was calculated for grouped VT and NVT serotypes. The estimate of post-PCV IPD incidence was calculated by multiplying these values with pre-PCV VT/NVT IPD proportions. IRRs were calculated for overall IPD and VT/NVT disease. Confidence intervals were calculated by Monte Carlo resampling. VE was calculated as 100 × (one minus the IRR). NT colonisation data were excluded from these models, since these pneumococci are rarely associated with IPD. To assess the impact of capture of multiple serotype colonisation data, separate models were fitted to simple dominant serotype colonisation data, latex sweep (phenotypic) multiple serotype colonisation data, and microarray (genotypic) multiple serotype colonisation data. The denominators used for these multi-serotype models were the number of swabs included in the analysis divided by the overall pneumococcal colonisation prevalence for the survey from which the swabs were selected (e.g. if 100 pneumococcus positive swabs were selected from the January 2018 survey, where the overall colonisation prevalence was 68.2%, then the denominator for the multi-serotype modelling would be 100/0.682 = 147).

To account for roll out of vaccine without a catch-up campaign, the post-PCV (2016-2018) period was split into early (2016-2017) and late (2018) phases. Colonisation and IPD data from 2015, the transition year from pre- to post-PCV eras, were not included in estimates of VE.

Tables

Table S1. Pneumococcal colonisation stratified by age category, pneumococcal serotype category, and time period

Age group	Pre-PCV	Post-PCV			P-value
	2014[a]	2016	2017	2018	
**0-11m**	262	170	151	164	
Pneumococcus detected, n (%)	206 (78.6)	116 (68.2)	83 (55.0)	110 (67.1)	0.0003
VT pneumococci detected, n (%)	151 (57.6)	70 (41.2)	34 (22.5)	47 (28.7)	<0.0001
NVT pneumococci detected, n (%)	59 (22.5)	45 (26.5)	47 (31.1)	66 (40.2)	<0.0001
NT pneumococci detected, n (%)	14 (5.3)	6 (3.5)	3 (2.0)	12 (7.3)	0.7
Colonised by multiple serotypes, n (%)	28 (10.7)	8 (4.7)	1 (0.7)	18 (11.0)	0.4
Colonised by a PNSSP isolate, n (%)	173 (66.0)	102 (60.0)	64 (42.4)	78 (47.6)	<0.0001
Colonised by a MDR isolate, n (%)	155 (59.2)	82 (48.2)	55 (36.4)	73 (44.5)	0.0002
**12-23m**	189	137	120	131	
Pneumococcus detected, n (%)	127 (67.2)	107 (78.1)	69 (57.5)	100 (76.3)	0.6
VT pneumococci detected, n (%)	92 (48.7)	59 (43.1)	34 (28.3)	38 (29.0)	<0.0001
NVT pneumococci detected, n (%)	31 (16.4)	47 (34.3)	29 (24.2)	62 (47.3)	<0.0001
NT pneumococci detected, n (%)	8 (4.2)	5 (3.6)	9 (7.5)	7 (5.3)	0.4
Colonised by multiple serotypes, n (%)	9 (4.8)	6 (4.4)	3 (2.5)	12 (9.2)	0.2
Colonised by a PNSSP isolate, n (%)	111 (58.7)	89 (65.0)	60 (50.0)	68 (51.9)	0.07
Colonised by a MDR isolate, n (%)	101 (53.4)	79 (57.7)	49 (40.8_	57 (43.5)	0.02
**24-35m**	106	59	72	72	
Pneumococcus detected, n (%)	64 (60.4)	38 (64.4)	36 (50.0)	46 (63.9)	0.9
VT pneumococci detected, n (%)	49 (46.2)	29 (49.2)	19 (26.4)	21 (29.2)	0.003
NVT pneumococci detected, n (%)	11 (10.4)	11 (18.6)	17 (23.6)	26 (36.1)	<0.0001
NT pneumococci detected, n (%)	4 (3.8)	0 (0.0)	2 (2.8)	2 (2.8)	0.8
Colonised by multiple serotypes, n (%)	2 (1.9)	3 (5.1)	3 (4.2)	5 (6.9)	0.1
Colonised by a PNSSP isolate, n (%)	57 (53.8)	33 (55.9)	31 (43.1)	28 (38.9)	0.03
Colonised by a MDR isolate, n (%)	53 (50.0)	30 (50.8)	24 (33.3)	29 (40.3)	0.06
**36-47m**	85	47	62	51	
Pneumococcus detected, n (%)	50 (58.8)	31 (66.0)	29 (46.8)	34 (66.7)	0.9
VT pneumococci detected, n (%)	30 (35.3)	22 (46.8)	17 (27.4)	13 (25.5)	0.1
NVT pneumococci detected, n (%)	20 (23.5)	10 (21.3)	11 (17.7)	20 (39.2)	0.1
NT pneumococci detected, n (%)	6 (7.1)	0 (0.0)	2 (3.2)	1 (2.0)	0.1
Colonised by multiple serotypes, n (%)	7 (8.2)	2 (4.3)	1 (1.6	3 (5.9)	0.3
Colonised by a PNSSP isolate, n (%)	38 (44.7)	24 (51.1)	24 (38.7)	30(58.8)	0.3
Colonised by a MDR isolate, n (%)	33 (38.8)	22 (46.8)	22 (35.5)	28 (54.9)	0.2
**48-59m**	79	36	44	35	
Pneumococcus detected, n (%)	43 (54.4)	22 (61.1)	27 (61.4)	19 (54.3)	0.8
VT pneumococci detected, n (%)	21 (26.6)	11 (30.6)	11 (25.0)	11 (31.4)	0.8
NVT pneumococci detected, n (%)	20 (25.3)	10 (27.8)	16 (36.4)	9 (25.7)	0.6
NT pneumococci detected, n (%)	3 (3.8)	2 (5.6)	2 (4.5)	2 (5.7)	0.7
Colonised by multiple serotypes, n (%)	3 (3.8)	1 (2.8)	2 (4.5)	2 (5.7)	0.6
Colonised by a PNSSP isolate, n (%)	34 (43.0)	18 (50.0)	20 (45.5)	16 (45.7)	0.8
Colonised by a MDR isolate, n (%)	26 (32.9)	15 (41.7)	18 (40.9)	16 (45.7)	0.2

VT: vaccine type; NVT: non-vaccine type; NT: non-typeable; PNNSP: penicillin non-susceptible *S. pneumoniae;* MDR: multi-drug resistant. [a]January and August survey data combined.

Table S2. Isolate-level antimicrobial resistance data, by survey

Survey period	Survey month(s)	Isolates analysed	Isolate antimicrobial resistance summary, n resistant (%)							
			MDR	CRO	C	DA	E	P	SXT	TE
Pre-PCV	Jan-2014	327	241 (73.7)	60 (18.3)	40 (12.2)	132 (40.4)	171 (52.3)	265 (81.0)	240 (73.4)	287 (87.8)
	Aug-2014	212	150 (70.8)	34 (16.0)	32 (15.1)	80 (37.7)	107 (50.5)	175 (82.5)	148 (69.8)	185 (87.3)
Post-PCV	Aug-2015	280	188 (67.1)	72 (25.7)	26 (9.3)	85 (30.4)	127 (45.4)	219 (78.2)	203 (72.5)	233 (83.2)
	Jan-2016	334	244 (73.1)	138 (41.3)	53 (15.9)	118 (35.3)	171 (51.2)	282 (84.4)	256 (76.6)	296 (88.6)
	Jan-2017	250	176 (69.3)	52 (20.5)	32 (12.6)	96 (37.8)	123 (48.4)	207 (81.5)	175 (68.9)	202 (79.5)
	Jan-2018	352	225 (63.9)	39 (11.1)	39 (11.1)	118 (33.5)	166 (47.2)	231 (65.6)	250 (71.0)	285 (81.0)
P-value (trend)			0.02	0.4	0.7	0.2	0.3	<0.0001	0.6	0.004

MDR: multi-drug resistant; CRO: ceftriaxone; C: chloramphenicol; DA: clindamycin; E: erythromycin; P: penicillin; SXT: co-trimoxazole; TE: tetracycline.

Table S3. Modelled estimates of PCV13 effectiveness based on pre-PCV invasive pneumococcal disease (IPD) data and pre- and post-PCV introduction colonisation data

	IRR (95% CI)	Change in disease rate, % (95% CI)
**All IPD**		
Early (2016-2017)	0.745 (0.648 – 0.849)	-25.5 (-35.2 to -15.1)
Late (2018)	0.736 (0.599 – 0.916)	-26.4 (-39.7 to -9.6)
**VT IPD**		
Early (2016-2017)	0.703 (0.605 – 0.806)	-29.7 (-39.5 to -19.4)
Late (2018)	0.637 (0.534 – 0.769)	-36.3 (-46.6 to -23.1)
**NVT IPD**		
Early (2016-2017)	1.291 (1.055 – 1.587)	+29.1 (+5.5 to +58.7)
Late (2018)	2.014 (1.622 – 2.496)	+101.4 (+62.2 to +149.6)

IRR: Incidence rate ratio; VT: vaccine type; NVT: non-vaccine type.

Table S4. Estimates of PCV13 effectiveness based on pre-PCV invasive pneumococcal disease (IPD) data and a subset of pre- and post-PCV introduction colonisation data processed to detect multiple serotype colonisation

	IRR (95% CI)	Change in disease rate, % (95% CI)
**Latex sweep serotyping**		
All IPD		
Early (2016-2017)	0.752 (0.619 – 0.896)	-24.8 (-38.1 to -10.4)
Late (2018)	0.783 (0.624 – 0.973)	-21.7 (-37.6 to -2.7)
VT IPD		
Early (2016-2017)	0.720 (0.589 – 0.868)	-28.0 (-41.1 to -13.2)
Late (2018)	0.708 (0.553 – 0.895)	-29.2 (-44.7 to -10.5)
NVT IPD		
Early (2016-2017)	1.177 (0.912 – 1.533)	+17.7 (-8.8 to +53.3)
Late (2018)	1.765 (1.376 – 2.239)	+76.5 (+37.6 to +123.9)
**Microarray serotyping**		
All IPD		
Early (2016-2017)	0.751 (0.616 – 0.921)	-24.9 (-38.4 to -7.9)
Late (2018)	0.788 (0.623 – 1.006)	-21.2 (-37.7 to +0.6)
VT IPD		
Early (2016-2017)	0.720 (0.570 – 0.776)	-28.0 (-43.0 to -22.4)
Late (2018)	0.707 (0.560 – 0.912)	-29.3 (-44.0 to -8.8)
NVT IPD		
Early (2016-2017)	1.153 (0.851 – 1.534)	-15.3 (-14.9 to +53.4)
Late (2018)	1.849 (1.394 – 2.437)	-84.9 (+39.4 to +143.7)

IRR: incidence rate ratio; CI: confidence interval; VT: vaccine type; NVT: non-vaccine type.

Figures

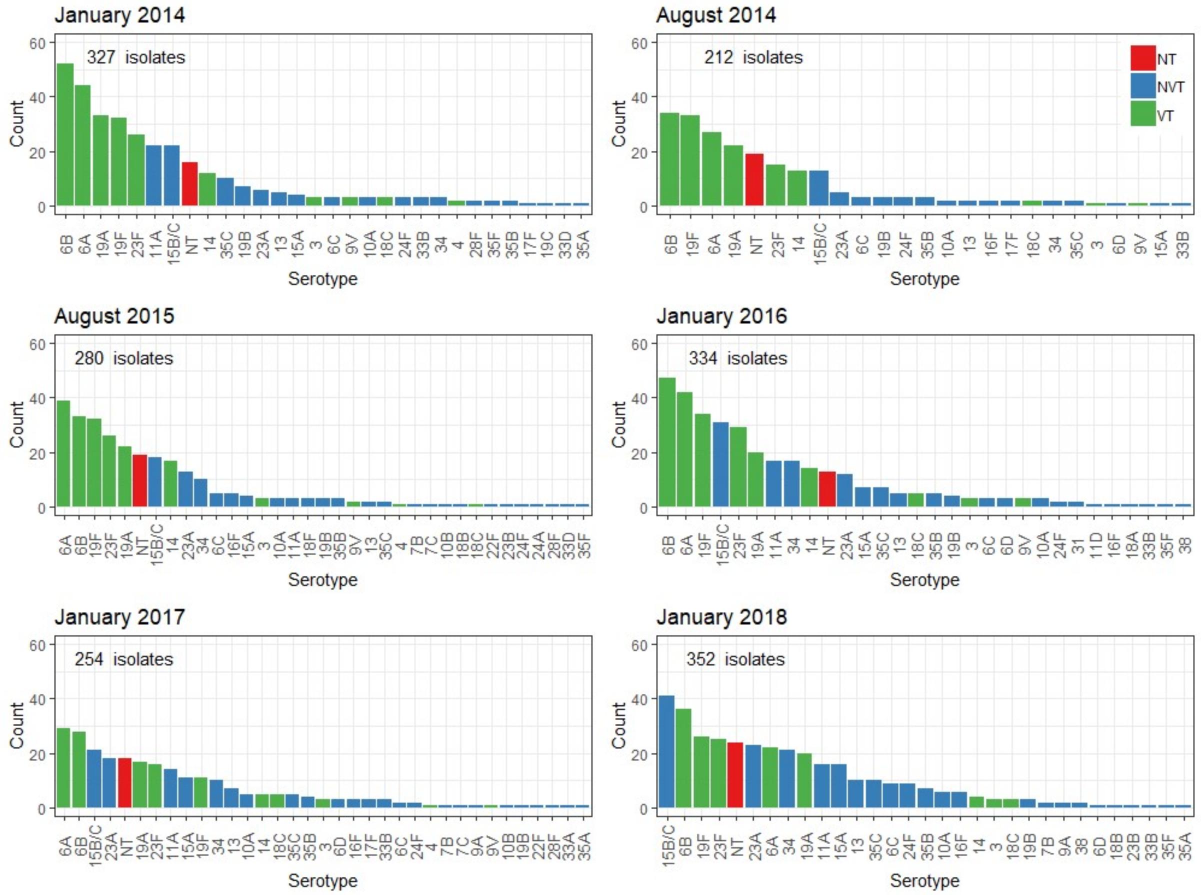

Figure S1. Pneumococcal isolates by colonisation survey, ordered by count and coloured by serotype category

VT: vaccine type; NVT: non-vaccine type; NT: non-typeable.

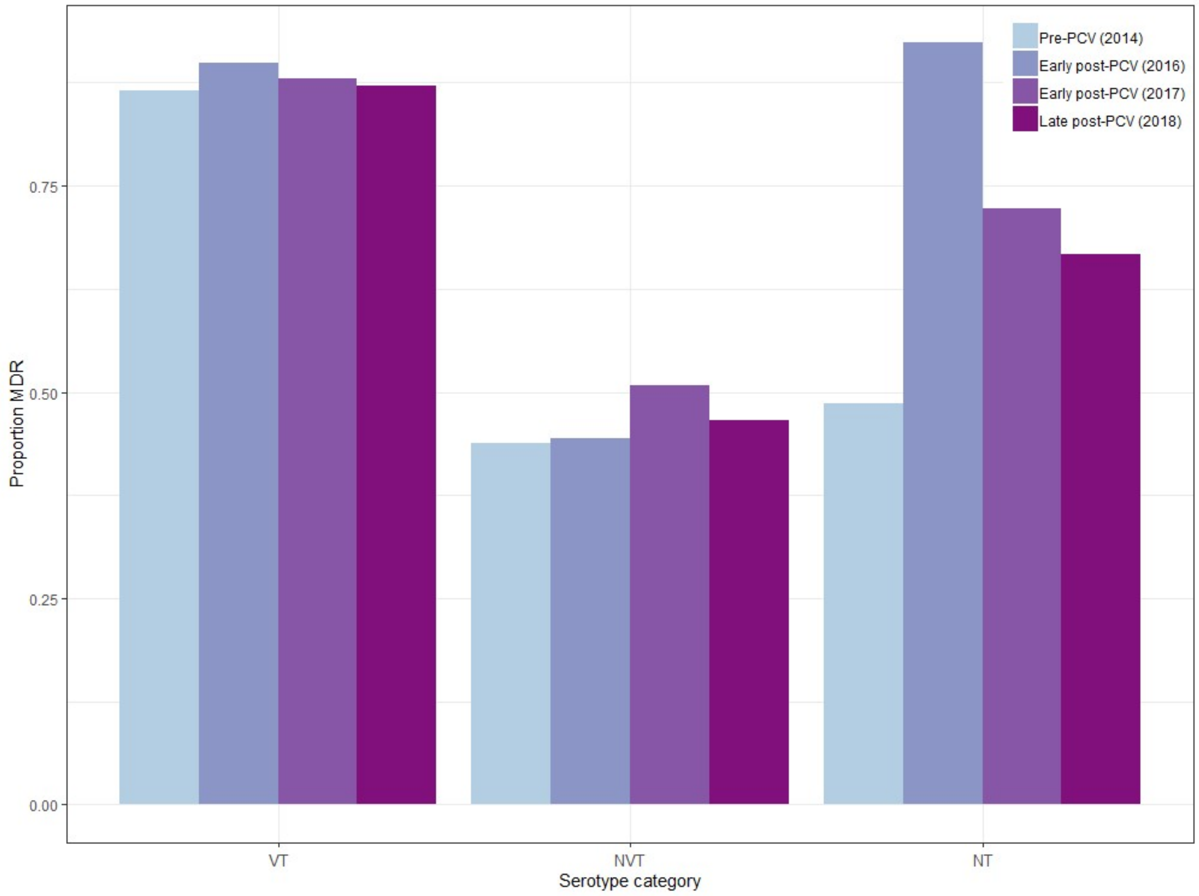

Figure S2. Proportion of pneumococcal isolates that were multi-drug resistant (MDR), by serotype category and time period

VT: vaccine type; NVT: non-vaccine type; NT: non-typeable.

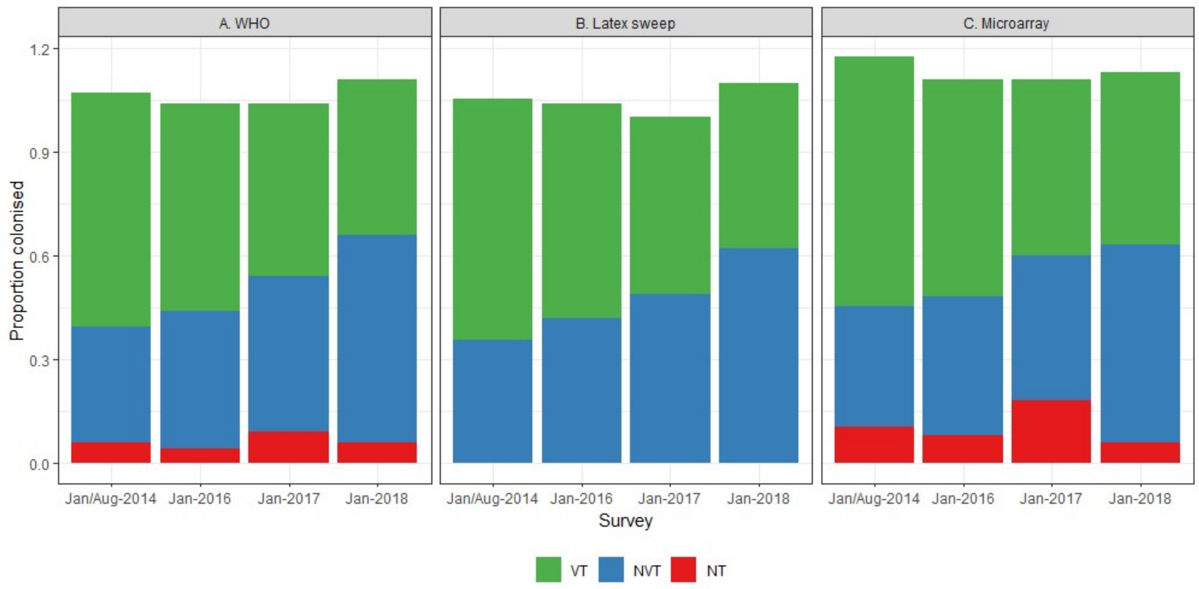

Figure S3. Colonisation by PCV13 serotypes, non-vaccine serotypes and non-typeable pneumococci for the 500 children included in the multiple colonisation work, by survey date and serotyping methodology

VT: vaccine type; NVT: non-vaccine type; NT: non-typeable. Overall proportions of children colonised are >1 as a result of multiple serotype detections. Latex sweep serotyping does not detect NT pneumococci.

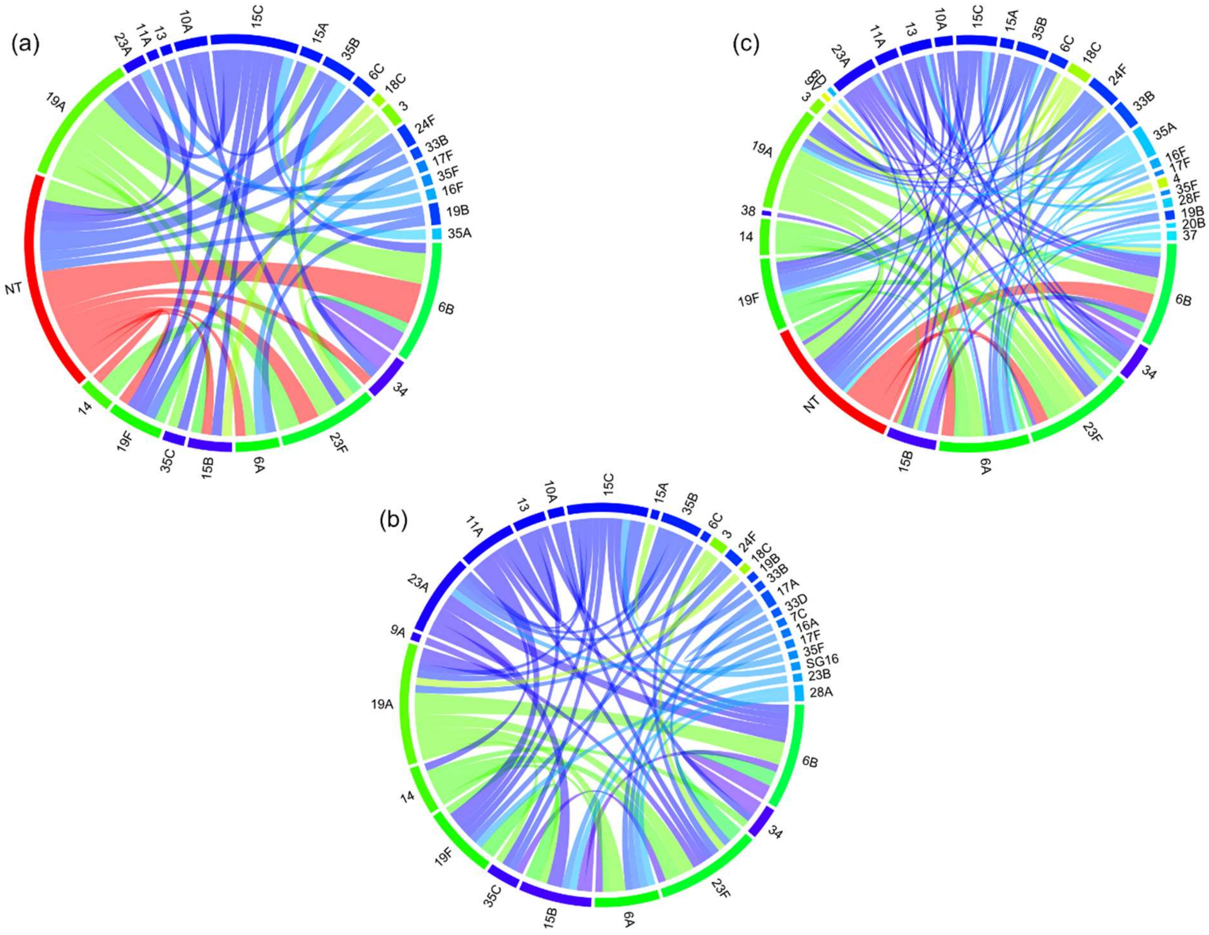

Figure S4. Chord plots summarising pneumococcal serotype co-colonisation patterns for the 500 children included in the multiple colonisation work, by serotyping methodology

(a) WHO serotyping methodology, (b) Latex sweep serotyping, (c) Molecular serotyping by microarray. Each serotype is represented around the edge of the chart. Co-detection with another serotype is indicated by a connecting line. The width of the lines is proportional to the number of serotype detections. VT serotypes are shaded green, NVT serotypes are shaded blue, and NT isolates are red (not detectable by latex sweep).

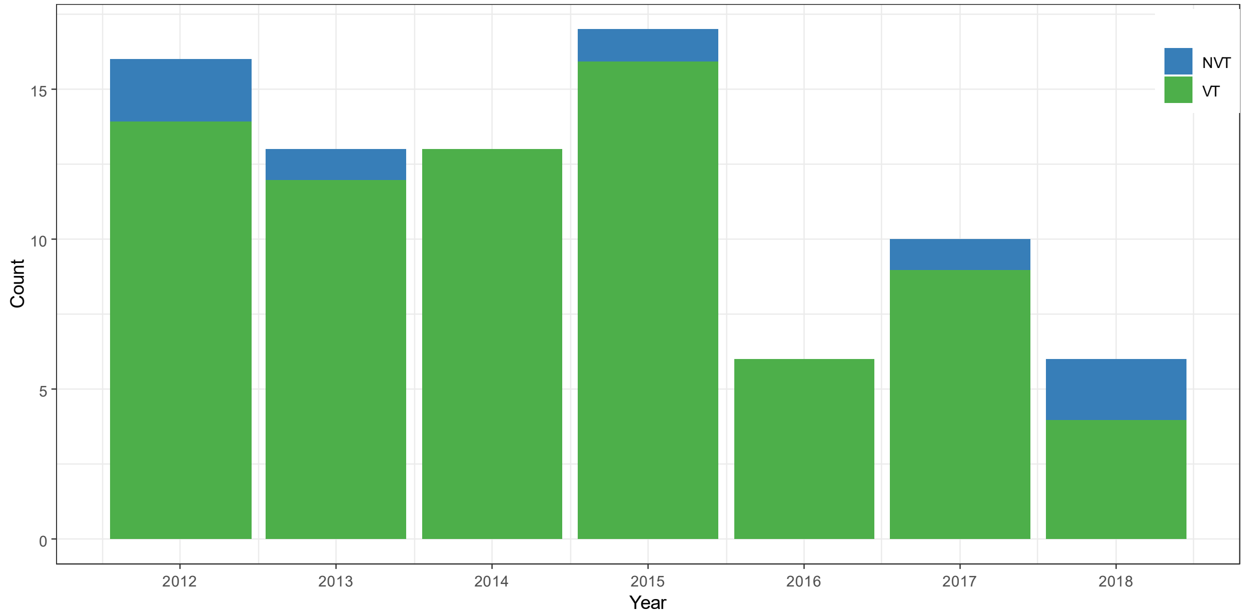

Figure S5. Invasive pneumococcal disease cases per year, in children <5 years admitted to Angkor Hospital for Children (1st January 2012 – 31st August 2018)

VT: vaccine type; NVT: non-vaccine type.

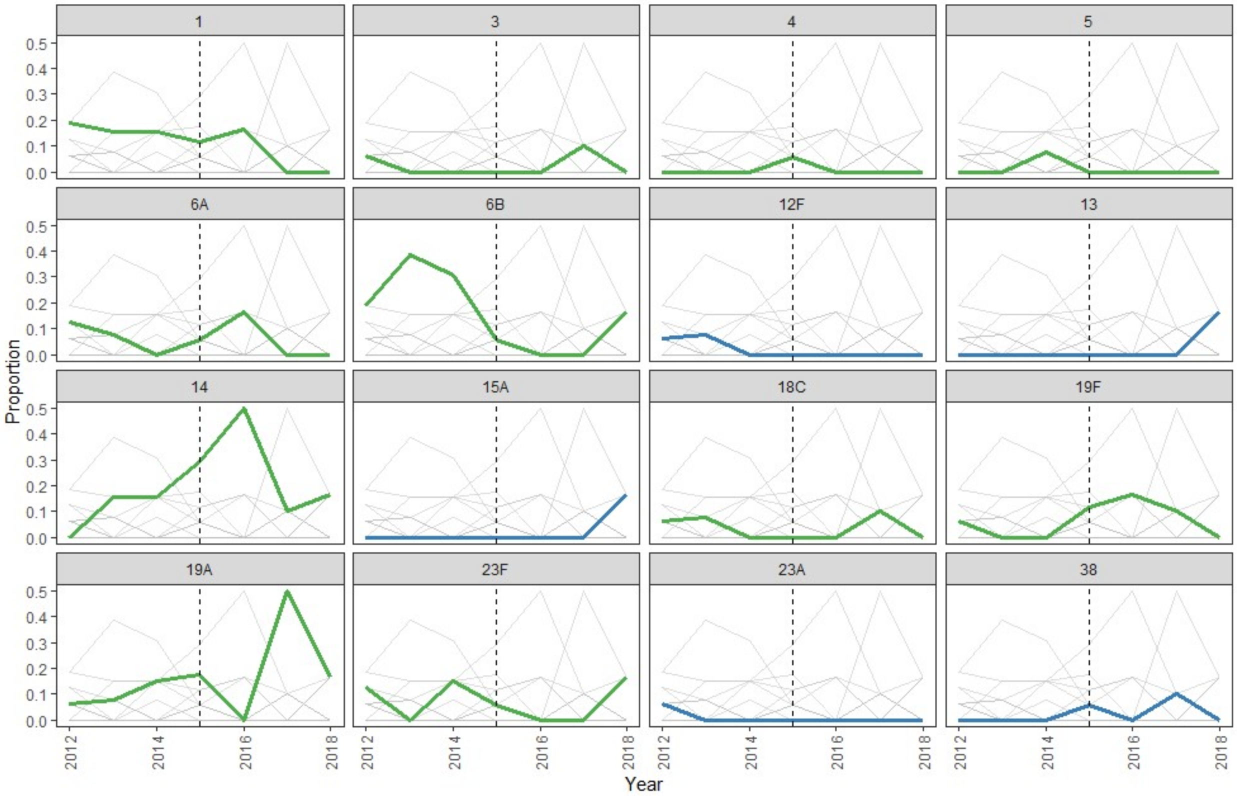

Figure S6. Invasive pneumococcal serotype distribution, proportions by vaccine period

Light grey lines show detected serotypes as a proportion of all isolates from that year, with a single serotype highlighted in colour (green = vaccine type; blue = non-vaccine type). The vertical black dashed line represents PCV13 introduction.

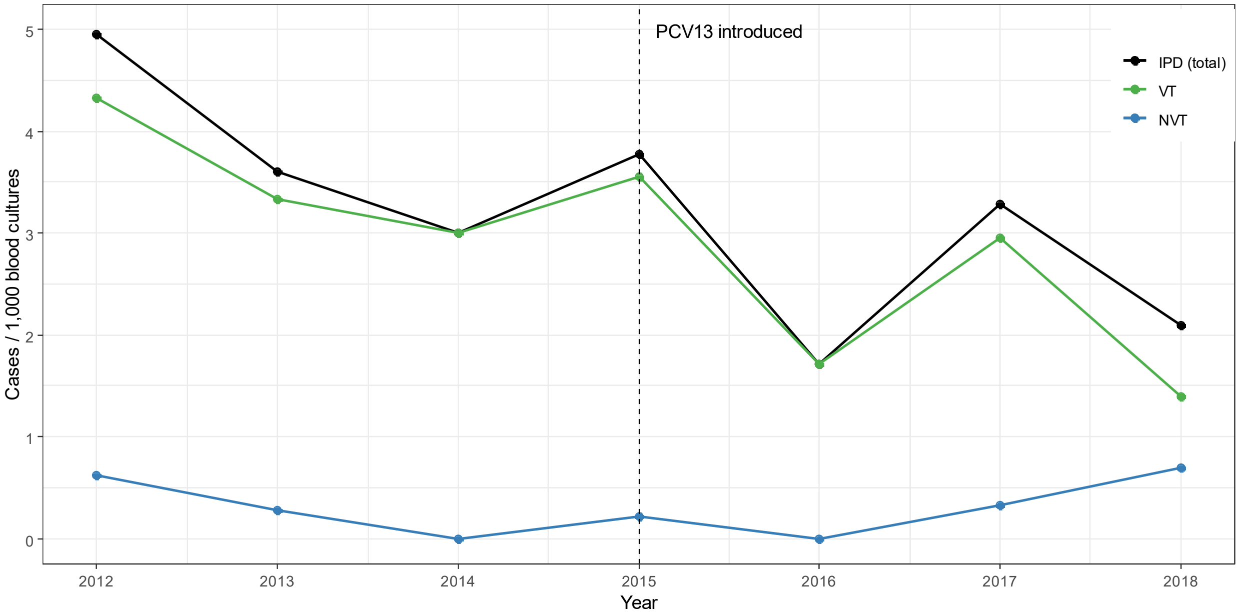

Figure S7. Invasive pneumococcal disease detection rate by time period, in children <5 years admitted to Angkor Hospital for Children (1st January 2012 – 31st August 2018)

VT: vaccine type; NVT: non-vaccine type.

References

1. Satzke C, Turner P, Virolainen-Julkunen A, et al. Standard method for detecting upper respiratory carriage of *Streptococcus pneumoniae*: updated recommendations from the World Health Organization Pneumococcal Carriage Working Group. Vaccine **2014**; 32(1): 165-79.

2. Turner P, Turner C, Suy K, et al. Pneumococcal Infection among Children before Introduction of 13-Valent Pneumococcal Conjugate Vaccine, Cambodia. Emerg Infect Dis **2015**; 21(11): 2080-3.

3. CLSI. Performance Standards for Antimicrobial Susceptibility Testing. CLSI document M02-A13. 13th Edition ed. Wayne, PA: Clinical and Laboratory Standards Institute, **2018**.

4. CLSI. Performance Standards for Antimicrobial Susceptibility Testing. CLSI document M100-S28. 28th Edition ed. Wayne, PA: Clinical and Laboratory Standards Institute, **2018**.

5. Russell FM, Biribo SS, Selvaraj G, et al. As a bacterial culture medium, citrated sheep blood agar is a practical alternative to citrated human blood agar in laboratories of developing countries. J Clin Microbiol **2006**; 44(9): 3346-51.

6. Yeh E, Pinsky BA, Banaei N, Baron EJ. Hair sheep blood, citrated or defibrinated, fulfills all requirements of blood agar for diagnostic microbiology laboratory tests. PLoS One **2009**; 4(7): e6141.

7. Satzke C, Seduadua A, Chandra R, Carapetis JR, Mulholland EK, Russell FM. Comparison of citrated human blood, citrated sheep blood, and defibrinated sheep blood Mueller-Hinton agar preparations for antimicrobial susceptibility testing of *Streptococcus pneumoniae* isolates. J Clin Microbiol **2010**; 48(10): 3770-2.

8. Turner P, Hinds J, Turner C, et al. Improved detection of nasopharyngeal cocolonization by multiple pneumococcal serotypes by use of latex agglutination or molecular serotyping by microarray. J Clin Microbiol **2011**; 49(5): 1784-9.

9. Satzke C, Dunne EM, Porter BD, Klugman KP, Mulholland EK, PneuCarriage project group. The PneuCarriage Project: A Multi-Centre Comparative Study to Identify the Best Serotyping Methods for Examining Pneumococcal Carriage in Vaccine Evaluation Studies. PLoS Med **2015**; 12(11): e1001903.

10. Dunne EM, Murad C, Sudigdoadi S, et al. Carriage of *Streptococcus pneumoniae, Haemophilus influenzae, Moraxella catarrhalis,* and *Staphylococcus aureus* in Indonesian children: A cross-sectional study. PLoS One **2018**; 13(4): e0195098.

11. Weinberger DM, Bruden DT, Grant LR, et al. Using pneumococcal carriage data to monitor postvaccination changes in invasive disease. Am J Epidemiol **2013**; 178(9): 1488-95.
